# Design and application of a lactulose biosensor

**DOI:** 10.1038/srep45994

**Published:** 2017-04-07

**Authors:** Jieyuan Wu, Peixia Jiang, Wei Chen, Dandan Xiong, Linglan Huang, Junying Jia, Yuanyuan Chen, Jian-Ming Jin, Shuang-Yan Tang

**Affiliations:** 1 CAS Key Laboratory of Microbial Physiological and Metabolic Engineering, Institute of Microbiology, Chinese Academy of Sciences, Beijing 100101, China; 2 Beijing Key Laboratory of Plant Resources Research and Development, Beijing Technology and Business University, Beijing 100048, China; 3 Core Facility for Protein Research, Institute of Biophysics, Chinese Academy of Sciences, Beijing 100101, China; 4 University of Chinese Academy of Sciences, Beijing 100049, China

## Abstract

In this study the repressor of *Escherichia coli lac* operon, LacI, has been engineered for altered effector specificity. A LacI saturation mutagenesis library was subjected to Fluorescence Activated Cell Sorting (FACS) dual screening. Mutant LacI-L5 was selected and it is specifically induced by lactulose but not by other disaccharides tested (lactose, epilactose, maltose, sucrose, cellobiose and melibiose). LacI-L5 has been successfully used to construct a whole-cell lactulose biosensor which was then applied in directed evolution of cellobiose 2-epimerase (C2E) for elevated lactulose production. The mutant C2E enzyme with ~32-fold enhanced expression level was selected, demonstrating the high efficiency of the lactulose biosensor. LacI-L5 can also be used as a novel regulatory tool. This work explores the potential of engineering LacI for customized molecular biosensors which can be applied in practice.

Transcription factor-based small-molecule biosensors have many potential applications in metabolic engineering for the overproduction of high-value small molecules through directed evolution^1^ or dynamic control of synthetic pathways^2^. However, the lack of available regulatory proteins that respond to small molecules of interest has limited the applications of these biosensors. Engineering the effector specificities of regulatory proteins is an efficient way to develop customized small-molecule biosensors as well as novel gene switches. Some regulatory proteins have been engineered to response to compounds other than their natural inducers^3^,^4^,^5^, however, only a few have been confirmed as functional *in vivo* biosensors in practice^6^,^7^,^8^. The specificity, sensitivity and robustness are important features for high-quality *in vivo* biosensors.

The *E. coli lac* regulatory system has been studied intensively for the past fifty years. The *lac* operon, which is required for the transport and metabolism of lactose, is controlled by LacI. In the absence of inducer, LacI binds the *lac* operator sequences within the *lac* operon, and transcription from the *lac* promoter is repressed. LacI undergoes a conformational change upon binding the inducer, after which its affinity for the operator sequence diminishes, and transcription from the *lac* promoter increases^9^,^10^. The direct inducer that binds to LacI is allolactose (6-*O*-β-d-galactopyranosyl-α-d-glucopyranose), the transgalactosylation product of β-galactosidase on lactose (4-*O*-β-d-galactopyranosyl-α-d-glucopyranose)^10^. Isopropyl 1-thio-β-d-galactoside (IPTG) is widely used as a gratuitous inducer of LacI.

Many mutagenesis studies have explored the relationship between the structure and function of LacI^11^,^12^,^13^,^14^,^15^,^16^,^17^. The crystal structure of LacI revealed the protein to be a homotetramer. Each subunit contains an N-terminal DNA binding domain, a core domain responsible for inducer binding, and a C-terminal domain comprising the tetramer interface^18^,^19^,^20^,^21^. The allosteric behavior of LacI has been studied extensively upon binding the inducer, the O6 hydroxyl of the galactoside is essential for establishing a water-mediated hydrogen bonding network that leads to a conformational change vital for allosteric transition^21^,^22^,^23^,^24^,^25^,^26^,^27^,^28^,^29^.

Lactulose is an important prebiotic ingredient in functional food. So far there have been two studies on lactulose biosensor construction, all based on the two enzymes fructose dehydrogenase (FDH) and β-galactosidase (β-gal). These biosensors were all used *in vitro*. Lactulose can be hydrolyzed to d-fructose and d-galactose by β-gal. In one study, the fructose was then oxidized by FDH which was simultaneously converted to its reduced form FDH-PQQH_2_. FDH-PQQH_2_ can be directly reoxidized by tetrathiafulvalen-tetracyanoquinodimetane (TTF-TCNQ) salt on a ring electrode, whose current was then monitored at 200 mV vs Ag/AgCl. 1.0 μM Lactulose can be detected by this lactulose sensor^30^. In the second case, the amount of fructose produced was measured with an electrochemical biosensor based on FDH, K_3_[Fe(CN)_6_] as mediator and a platinum based electrochemical transducer^31^.

In this study, the effector-binding pocket of LacI was engineered using directed evolution strategy. A LacI mutant (LacI-L5) with the ability to be specifically induced by the altered effector, lactulose, was obtained. LacI-L5 has been successfully applied as a lactulose biosensor in engineering cellobiose 2-epimerase (C2E)^32^ for enhanced lactulose synthesis efficiency. This work revealed the potential of engineering LacI for customized molecular biosensors which can be applied in practice.

## Results

### Construction of LacI mutagenesis library

**Figure 1:**
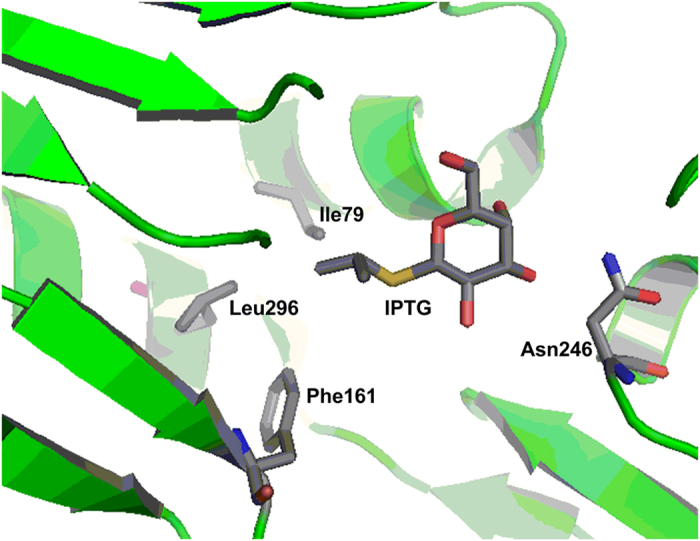
Crystal structure of the wild-type LacI binding pocket in complex with IPTG (PDB ID: 2P9H)^21^, based on which four residues in the effector binding pocket were selected for saturation mutagenesis.

Based on the crystal structure of LacI in complex with its gratuitous inducer, IPTG^21^, the amino acid residues I79, F161, N246, L296 were chosen as targets of saturation mutagenesis. Three of these residues, I79, F161 and L296, are located in the binding pocket of the reducing-end sugar residue, while N246 is located in the binding pocket for the non-reducing-end sugar residue (Fig. 1). A library of 1.08 × 10^6^ LacI mutants, consisting of all possible mutations, was constructed and subjected to Fluorescence Activated Cell Sorting (FACS) screening. The library was expressed in the BLGR strain, in which wild-type *lacI* is deficient.

### Screening the library for LacI mutants with altered effector specificity

**Figure 2:**
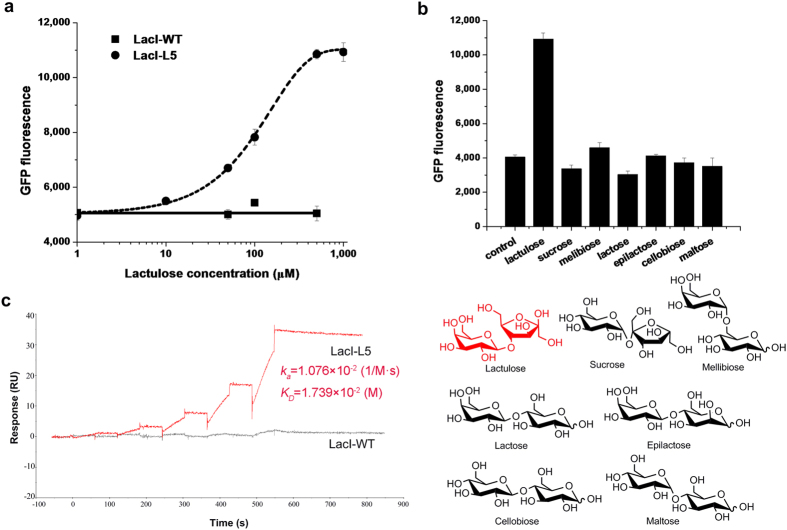
Engineering LacI for altered effector specificity. (**a**) GFP fluorescence from strain BLGR harboring plasmid pLac7-L5 as a function of lactulose. The corresponding dose-response curve of wild-type LacI (LacI-WT) is included for reference. (**b**) GFP fluorescence from the strain BLGR, which harbors plasmid pLac7-L5, in response to 1 mM of the indicated disaccharides, and the structures of the disaccharides. (**c**) The binding affinity of LacI-L5 for lactulose was measured by SPR. The corresponding binding affinity of wild-type LacI (LacI-WT) is included for reference.

To test the feasibility of engineering LacI for altered effector specificity, positive screening was performed in the presence of 10 mM lactulose (4-*O*-β-d-galactopyranosyl-d- fructofuranose) and negative screening was performed in the presence of 10 mM lactose or in the absence of inducer, in order to eliminate the LacI mutants responsive to lactose or with high leaky expression. After four rounds of the dual screening, eight mutants displaying responses to lactulose were selected, among which the mutant LacI-L5, carrying one amino acid substitution (F161K), showed ~2.7-fold induction of GFP expression in response to 10 mM lactulose, while wild-type LacI did not respond to lactulose at all (Figure S1a). The lactulose dose-response curves of LacI-L5 and wild-type LacI are shown in Fig. 2a. LacI-L5 exhibited an approximately 2.7-fold induction of GFP expression over a lactulose concentration range of 0.001–1 mM. Half-maximal induction occurs at 0.13 mM lactulose.

### Characterization of the LacI-L5 mutant

The induction effect of several commercially available disaccharides, including lactose, maltose, sucrose, cellobiose, melibiose and epilactose, on LacI-L5 was further assessed, revealing almost no induction in LacI-L5 (Fig. 2b). However, LacI-L5 remained responsive to IPTG induction, although to a less extent than wild-type LacI (Figure S1b). LacI-L5 and wild-type LacI were purified and their binding affinities for lactulose were measured by surface plasmon resonance (SPR). The results showed that lactulose was bound to LacI-L5 with a *K*_*D*_ of 17 mM, while it was not bound to wild-type LacI at all (Fig. 2c).

**Figure 3:**
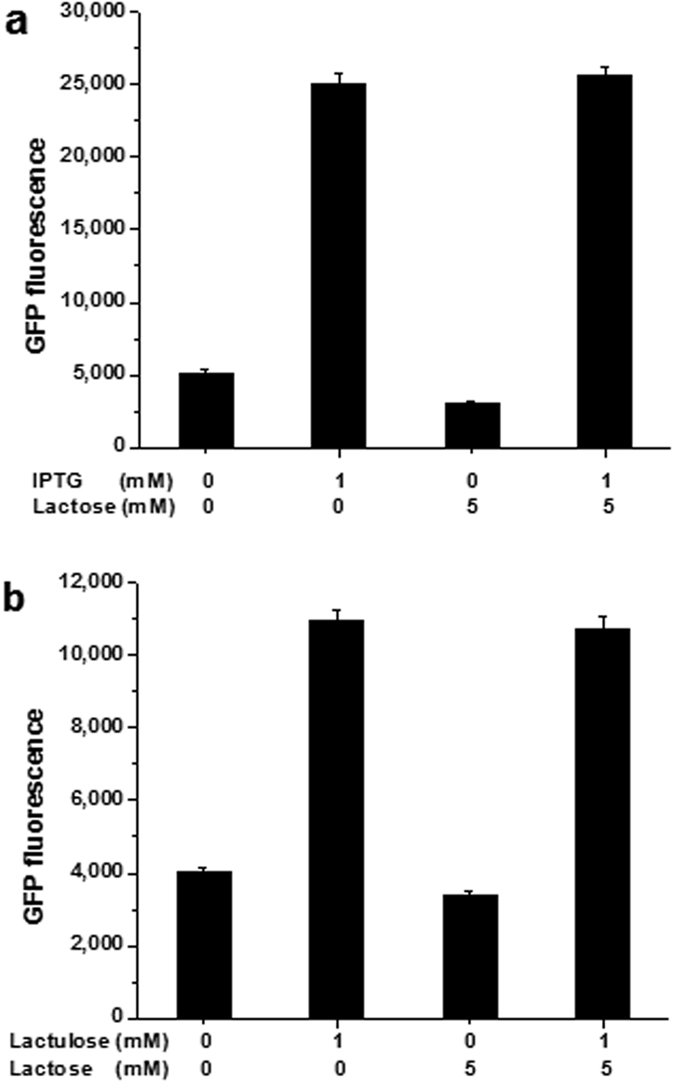
Effects of lactose on wild-type LacI (**a**) and LacI-L5 (**b**).

Because lactose, whose binding increases the affinity of the *lac* repressor for the operator, acts as an anti-inducer of wild-type LacI^33^, its effects on LacI-L5 was studied. For LacI-L5, the anti-inducing effect of lactose remained similar to that in wild-type LacI, however, the induction of LacI-L5 by lactulose was not inhibited by lactose (Fig. 3).

### Application of LacI-L5 in engineering C2E for elevated lactulose production

**Figure 4:**
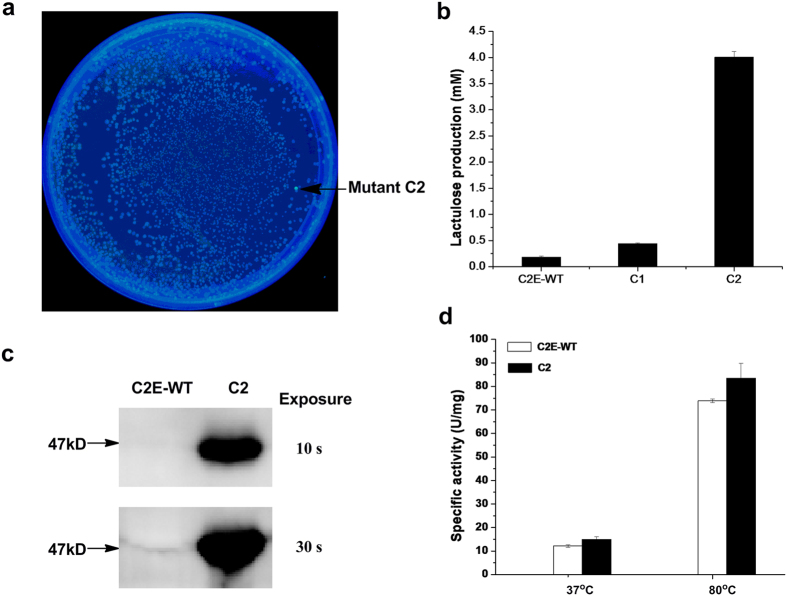
Engineering C2E for elevated lactulose production. (**a**) The BLGR strains co-expressing LacI-L5 and the C2E random mutagenesis library grown on LB agar supplemented with 10 mM lactose under UV 302 nm. (**b**) Lactulose productions of the selected variants expressing the C2E mutants at 37 °C, compared with that of strain expressing the wild-type C2E (C2E-WT). (**c**) Western-blot analysis of mutant C2 expression as compared with the wild-type enzyme. (**d**) Specific activities of wild-type C2E (C2E-WT) and mutant C2 at 37 °C and 80 °C.

To test the effectiveness of strain BLGR harboring plasmid pLac7-L5 as a whole-cell biosensor of lactulose, it was used as a high-throughput screening tool for engineering C2E from *Caldicellulosiruptor saccharolyticus*^32^ for improved lactulose production. The plasmid pN25-C2E harboring the C2E random mutagenesis library was co-transformed with the plasmid pLac7-L5 into strain BLGR. Lactulose endogenously produced by C2E mutants was found to bind to LacI-L5 and activate the expression of GFP inside the cell. Two colonies, expressing mutant enzymes C1 and C2, showing stronger GFP fluorescence than colonies expressing wild-type C2E on LB agar supplemented with lactose, were selected from the library (Fig. 4a). Because LacI-L5 was not responsive to lactose, any fluorescence expression would be induced by the lactulose produced by C2E mutants inside the cells using lactose as the substrate. HPLC analyses verified enhanced lactulose production by these selected variants compared with that of the strain expressing wild-type C2E. The best of the variants, C2(E272D, F339S), displayed ~22-fold increase in lactulose production at 37 °C (Fig. 4b). Western blot analysis revealed that the expression level of mutant C2 was significantly elevated by ~32-fold, compared with that of the wild-type enzyme (Fig. 4c), although no obvious increase in codon usage bias was observed in the mutations in mutant C2. The pN25-C2E plasmid expressing mutant C2 was isolated and retransformed into strain BLGR, and a significantly elevated expression level of the mutant C2E was confirmed.

For further characterization, mutant C2 was purified as a C-terminal His-tagged fusion protein for use in activity assay along with the wild-type enzyme. The specific activity of purified mutant C2 displayed moderate increases of 1.3- and 1.1-fold at 37 °C and 80 °C (optimum reaction temperature of wild-type C2E), respectively, indicating that the significantly elevated expression level of mutant C2 was the main factor underlying the enhanced lactulose production in the selected hyper-producing strain (Fig. 4d). In addition, C2E was reported to simultaneously produce lactulose and epilactose from lactose^32^ (Figure S2a). Epilactose production in the reaction mixtures was further explored, and at 37 °C, mutant C2 displayed an increase in productivity in producing epilactose similar to that for lactulose (Figure S2b).

## Discussion

Wild-type LacI displays a stringent induction specificity for allolactose. Apart from allolactose and IPTG, an IPTG analog, n-propyl 1-thio-β-d-galactoside, was reported to exhibit a comparative inducing effect on LacI^33^. Attempts have been made to engineer it to response to compounds other than its natural inducers, but high induction specificity is still a challenge^5^. The LacI-L5 mutant obtained here exhibited responsiveness towards lactulose but was not induced by other six disaccharides tested, among which the structures of lactose, melibiose and epilactose only differ from lactulose in the sugar residue at the reducing end. Thus, the effector-binding pocket showed relatively good specificity towards lactulose. We found that the inducing effect of IPTG was difficult to ablate, even by FACS negative screening in the presence of IPTG, probably due to the relatively smaller steric effect of the isopropyl group. Our work revealed that the effector specificity of LacI has the potential to be engineered towards various sugars as well as other similar small molecules, and these LacI mutants will have wide applications as novel regulatory tools and as customized sensors for small-molecule products.

As a whole-cell biosensor, strain BLGR harboring plasmid pLac7-L5 was successfully used for high-throughput screening of the C2E mutagenesis library for lactulose hyper-producers. Interestingly, for C2E mutant C2, the ~32-fold increase in expression level seemed not to be due to its higher codon usage bias, which is thought to affect expression levels via the abundance of various tRNA molecules^34^,^35^. Additional factors influencing expression level include mRNA secondary structure and protein stability^36^,^37^,^38^. Thus, in addition to improving enzyme activity, *in vivo* directed evolution is a powerful tool for engineering protein expression level and protein solubility, all of which require *in vivo* high-throughput screening tools.

Lactulose is an important prebiotic ingredient in functional food^39^,^40^. Therefore, the C2E mutants obtained in this study have potential applications in the industrial production of this molecule.

In conclusion, the *lac* repressor, LacI, has been engineered to response to lactulose, based on which a whole-cell lactulose biosensor was developed and successfully applied in high-throughput screening of lactulose hyper-producing strains. This work has revealed the potential of engineering the *lac* repressor for customized *in vivo* small-molecular biosensors and novel gene switches.

## Methods

### Strain construction and culture conditions

The *lacI, galK* and *recA* in strain *E. coli* BW25113 was deleted via P1 phage transduction using phage libraries of genomic DNA from strain JW0336-1, JW0740-3 and JW2669-1, respectively^41^. The deletion was verified by PCR and the FRT-flanked *kan* gene was then removed using flipase-mediated recombination^42^, resulting in strain BLGR.

All bacteria were routinely grown in Luria-Bertani (LB) medium (10 g·L^−1^ tryptone, 5 g·L^−1^ yeast extract, 5 g·L^−1^ NaCl). The antibiotics ampicillin (100 μg·mL^−1^) and kanamycin (50 μg·mL^−1^) were used when necessary.

### Plasmid construction

All strains and plasmids used in this study are shown in Table S1. Sequences for all primers are listed in Table S2. Gene *gfpuv* was amplified from plasmid pBAD-GFPuv (Clontech) using primers gfp-for-AseI and gfp-rev-XbaI, and the PCR product was subsequently ligated into vector pFLAG-CTC (Sigma-Aldrich), resulting in plasmid pLac7 (*gfpuv* gene expression is controlled by a *tac* promoter). The gene encoding C2E was amplified using the genomic DNA of strain *C. saccharolyticus* DSM 8903 as template with primers C2E-KpnI-for and C2E-SacI-rev, and the fragment was ligated with the PCR product amplified with primers pBADLacS-R-SacI-for and pBADLacS-R-KpnI -rev using plasmid pBADLacS-R as template, after digestion with SacI and KpnI, resulting in plasmid pBADC2E carrying the gene encoding C-terminal 6×His-tagged C2E. Promoter pN25 (kindly provided by Prof. Chunbo Lou from Institute of Microbiology, Chinese Academy of Sciences) was amplified with primers pN25-SphI-for and pN25-SpeI-rev, and the PCR product was ligated into plasmid pBADC2E after digestion with SphI and SpeI, resulting in plasmid pN25-C2E.

For purification of wild-type and mutant LacIs, the wild-type or mutant LacI gene was amplified with primers LacI-Ex-NdeI-for and LacI-Ex-XhoI-rev using plasmid pLac7, and pLac7-L5 as template, and then ligated into pBADLacS^43^ after digestion with NdeI and XhoI, resulting in plasmid pBADLacI expressing wild-type or mutant LacI under the control of promoter P_BAD_.

### LacI library construction

LacI site-directed saturation library was constructed with overlap extension PCR method. Fragments of *lacI* (1, 2 and 3) were amplified using plasmid pLac7 as template with the following primer sets: LacI-I79-for and LacI-F161-rev, LacI-F161-for and LacI-N246-rev, LacI-N246-for and LacI-L296-rev. The Fragments were gel-purified and equimolar aliquots of adjacent fragments were combined (1+2; 2+3) and PCR-assembled without primers. Finally, the two assemblies were combined and outer primers LacI-I79-for and LacI-L296-rev were added to perform PCR. The PCR product containing all four mutagenesis sites (79, 161, 246 and 296) was used as megaprimer to perform MEGAWHOP PCR^44^. DpnI digestion (20 U) of the template was performed at 37 °C for 2 h, the DpnI was inactivated at 80 °C for 20 min. The PCR product was transformed into *E. coli* MC1061 and around 1.08 × 10^6^ transformants were recovered. Ten randomly picked clones were sequenced and these sequences revealed the expected random mutations at the targeted nucleotide positions, with no additional point mutations. All colonies from the agar plates were used for plasmid isolation to prepare the plasmid library.

### LacI library screening

The LacI library was transformed into the BLGR strain, and 1.0 × 10^7^ transformants were recovered. Fluorescence-activated cell sorting (FACS) was performed using a FACS AriaII sorter (BD, San Jose, USA). Fluorescence was excited at 405 nm, and emission was collected using a 530/30 nm filter. In the first round of negative screening, cells were precultured overnight in LB medium and then diluted to OD_600_ = 0.2 in the same medium in the absence of lactulose. The cells were then grown for 12 h and the least fluorescent 8.0 × 10^5^ cells were collected from a total of 1 × 10^6^ cells (representing 80% of all cells sorted). This was performed to eliminate LacI variants with high levels of leaky expression. The collected cells showing low fluorescence were subjected to another negative screen in the presence of 10 mM lactose to eliminate clones that were induced by lactose. The collected cells were then induced with 10 mM lactulose and grown for 12 h to enable positive screening, and the most fluorescent 8.0 × 10^3^ cells were sorted from a total of 1 × 10^6^ cells. This procedure was repeated in a second round of negative and positive screens. This dual screening was repeated four times, followed by a final round of negative screening in the absence of inducer. Ten clones were selected for rescreening in the test tubes.

### Construction of the random mutagenesis library of C2E

The random mutagenesis library of C2E was constructed through error-prone PCR. Primer pairs C2E-for/C2E-rev were used to amplify the C2E gene, using plasmid pN25-C2E as template. The PCR reaction mixture consisted of 5 mM MgCl_2_, 0.2 mM each of dATP and dGTP, 1 mM each of dCTP and dTTP, 0.1 mM MnCl_2_ and rTaq DNA polymerase. Then the PCR products obtained, containing randomly mutated C2E gene, were used as megaprimer to perform MEGAWHOP PCR using plasmid pN25-C2E as template. Following the MEGAWHOP PCR, DpnI digestion (20 U) of the template was performed at 37 °C for 2 h. The DpnI was inactivated at 80 °C for 20 min. The PCR products were transformed into *E. coli* MC1061 and around 1 × 10^6^ transformants were recovered. Ten randomly picked clones from the library were sequenced and contained an average of 3 nucleotide mutations per clone. All colonies from the agar plates were used for plasmid isolation to prepare the plasmid library.

### Screening of the C2E random mutagenesis library

The C2E random mutagenesis library was transformed into the BLGR strain harboring the plasmid pLac7-L5. The cells were then grown on LB agar plates containing 10 mM lactose. After culturing for 12 h, mutant strains showing stronger GFP fluorescence than colonies expressing wild-type C2E were selected for rescreening in the test tubes.

### GFP fluorescence assays

A colony of strain BLGR harboring plasmid pLac7 expressing wild-type or mutant LacI was grown for 12 h in LB medium, then diluted to OD_600_ = 0.2 in the same medium containing appropriate concentrations of inducers, and allowed to grow under inducing condition for 12 h. A total of 200 μl of culture was centrifuged, and the cells were washed with 10 mM phosphate buffered saline (PBS, pH 7.4) and resuspended in 200 μl of the same buffer. The OD_600_ and fluorescence emission were measured with a SynergyMx Multi-Mode Microplate Reader (BioTek, Vermont, USA) (395 nm excitation filter and 509/20 nm emission filter). The background fluorescence due to buffer was subtracted in all measurements.

A colony of strain BLGR harboring plasmids pN25-C2E and pLac7-L5 was grown for 12 h in LB medium and then diluted to OD_600_ = 0.2 in the same medium containing 10 mM lactose, and allowed to grow for 12 h. 200 μl culture was collected and the cell density and GFP fluorescence were measured as described above.

All GFP fluorescence data shown in figures were normalized with respect to OD_600_. All reported data in Figs 2 and 3 and Figure S1 are presenting the mean of three independent data points. The error bars represent standard deviations.

### SPR assay

SPR measurements were performed on a Biacore T100 apparatus (GE healthcare, Uppsala, Sweden) at 25 °C. To measure the binding affinities of LacI-L5 and wild-type LacI for lactulose, purified LacI-L5 and wild-type LacI were immobilized covalently to the carboxyl groups in the dextran layer of the sensor chip CM5 (GE healthcare). Then, various concentrations of lactulose (0.3, 0.6, 1.2, 2.4 and 4.8 mM) were injected at a constant flow rate of 20 μl/min onto the dextran layers. PBS (pH 7.4) was used as the running buffer.

Data were collected with the Biacore control software. Experiments were performed by monitoring the refractive index changes as a function of time under constant flow conditions. The relative amount of binding was determined by measuring the net increase of refractive index over time compared with that of running buffer alone. There is an inline subtraction of reference surface during the run. Kinetic association (*k*_*a*_) and dissociation (*k*_*d*_) constants were calculated by Biacore T100 evaluation software 2.0.1. The equilibrium dissociation constant *K*_*D*_ values were calculated with the equation *K*_*D*_ = *k*_*d*_/*k*_*a*_.

### Activity assay

The production of lactulose was quantified by HPLC. For purified C2E, one unit (U) of enzyme activity was defined as the amount of enzyme required to produce 1 μmol lactulose from lactose per hour.

Data in Fig. 4d represent the mean of three independent data points. The error bars represent standard deviations.

### HPLC identification of the reaction products

The reaction mixture for lactulose production (500 μl) was constituted of 50 mM PIPES buffer (pH 7.5), 30 g∙L^−1^ lactose and 50 μl crude or purified C2E enzyme. The reaction was incubated at 37 °C or 80 °C for 12 h. The reaction was stopped by cooling at 4 °C instantly.

The lactulose produced by C2E was quantified by the HPLC system described above, with the mobile phase of 80% acetonitrile at a flow rate of 0.5 mL·min^−1^. The product epilactose was quantified by a DX-600 HPAEC system (Dionex 600, sunnylvale, CA) equipped with an electrochemical detector. Separation was achieved using a CarboPac PA-10 column with the mobile phase of 18 mM sodium hydroxide at a flow rate of 0.5 mL∙min^−1^.

All reported data in Fig. 4b and Figure S2b represent the mean of three independent data points. The error bars represent standard deviations.

## Additional Information

**How to cite this article**: Wu, J. *et al*. Design and application of a lactulose biosensor. *Sci. Rep.* **7**, 45994; doi: 10.1038/srep45994 (2017).

**Publisher's note:** Springer Nature remains neutral with regard to jurisdictional claims in published maps and institutional affiliations.

## Supplementary Material

Supplementary Information
