# Supplementary material for: Design and application of a lactulose biosensor
Source: Sci Rep. 2017 Apr 7;7:45994. doi: 10.1038/srep45994 (PMC5384092; doi:10.1038/srep45994)
Supplement: Supplementary Information [file srep45994-s1.pdf]

**Supplementary Information for**

# Design and application of a lactulose biosensor

Jieyuan Wu[1,4], Peixia Jiang[1], Wei Chen[1], Dandan Xiong[1,4], Linglan Huang[2], Junying Jia[3], Yuanyuan Chen[3], Jian-Ming Jin[2*] & Shuang-Yan Tang[1*]

[1]CAS Key Laboratory of Microbial Physiological and Metabolic Engineering, Institute of Microbiology, Chinese Academy of Sciences, Beijing 100101, China. [2]Beijing Key Laboratory of Plant Resources Research and Development, Beijing Technology and Business University, Beijing100048, China. [3]Core Facility for Protein Research, Institute of Biophysics, Chinese Academy of Sciences, Beijing 100101, China. [4]University of Chinese Academy of Sciences, Beijing 100049, China.

*Correspondence and requests for material should be addressed to S-Y. T (email: tangsy@im.ac.cn) or to J-M. J (email: jinjianming@btbu.edu.cn).

## Supplementary methods

**Materials.** Restriction enzymes and DNA Polymerase were purchased from TaKaRa (Dalian, China). DNA ligase and oligonucleotides were purchased from Life Technologies (Shanghai, China). Lactulose was purchased from Sigma-Aldrich (St.Louis, USA). Epilactose was purchased from Carbosynth Limited (Berkshire, UK). Other chemicals and reagents were of high quality and obtained from standard commercial sources. DNA sequencing was performed at Life Technologies (Shanghai, China). Strain *Caldicellulosiruptor saccharolyticus* DSM 8903 was kindly provided by Prof. Fuli Li from Qingdao Institute of Biomass Energy and Bioprocess Technology, Chinese Academy of Sciences.

**Purification of 6×His tagged wild-type LacI, C2E and their mutants.** A colony of strain BLGR harboring plasmid pN25-C2E expressing wild-type or mutant C2E was grown in 3 ml of LB at 37°C for 12 h. The culture was then diluted to $OD_{600}$=0.05 in 100 ml of the same medium and grown at 37°C for 14 h. Cells were harvested by centrifugation at 4°C at 5000 ×g for 10 min. The cells were then resuspended in 10 ml of 50 mM Tris-HCl buffer (pH 8.0, 300 mM NaCl, 20 mM imidazole) and lysed by sonication. The lysate was centrifuged at 10000 ×g for 10 min at 4°C. The supernatant was applied to Ni-NTA resin (Sigma-Aldrich). The column was washed with 50 mM Tris-HCl buffer (pH 8.0, 300 mM NaCl, 20 mM imidazole) and the bound protein was eluted with 50 mM Tris-HCl buffer (pH 8.0, 300 mM NaCl, 250 mM imidazole). The imidazole was removed by dialysis at 4°C against 50 mM PIPES buffer (pH 7.5).

A colony of strain BW25113 harboring plasmid pBADLacI expressing wild-type or mutant LacI was grown in 3 ml of LB at 37°C for 12 h. The culture was then diluted to $OD_{600}$=0.05 in 100 ml of the same medium and grown at

37°C and 1 mM L-arabinose was added when $OD_{600}$ reached 0.8, then the culture was continuously grown at 30°C for 12 h. The cells were harvested, lysed and the 6×His tagged LacI protein was purified as described above. The purified protein was dialyzed against 10 mM PBS (pH 7.4).

The purity of proteins were assessed with SDS-PAGE and the protein concentration was assayed with Bradford method.

**Western blot.** Equal amounts of crude protein extracted from strain BLGR expressing wild-type or mutant C2E were separated by SDS-PAGE and transferred to PVDF membrane (Millipore, Temecula, USA). The transfers were sequentially blocked with blocking buffer (5% skim milk powder in PBS) at 37°C for 1 h, incubated with mouse anti-His tag monoclonal antibody overnight at 4°C, and incubated with horseradish peroxidase-conjugated secondary antibodies. Each time after incubation with the antibodies, the membranes were washed three times (10 minutes per time) with PBS (pH 7.4, 0.05% Tween-20). Detection was conducted using Enhanced Chemiluminescence Western Blotting Detection Reagents (Millipore).

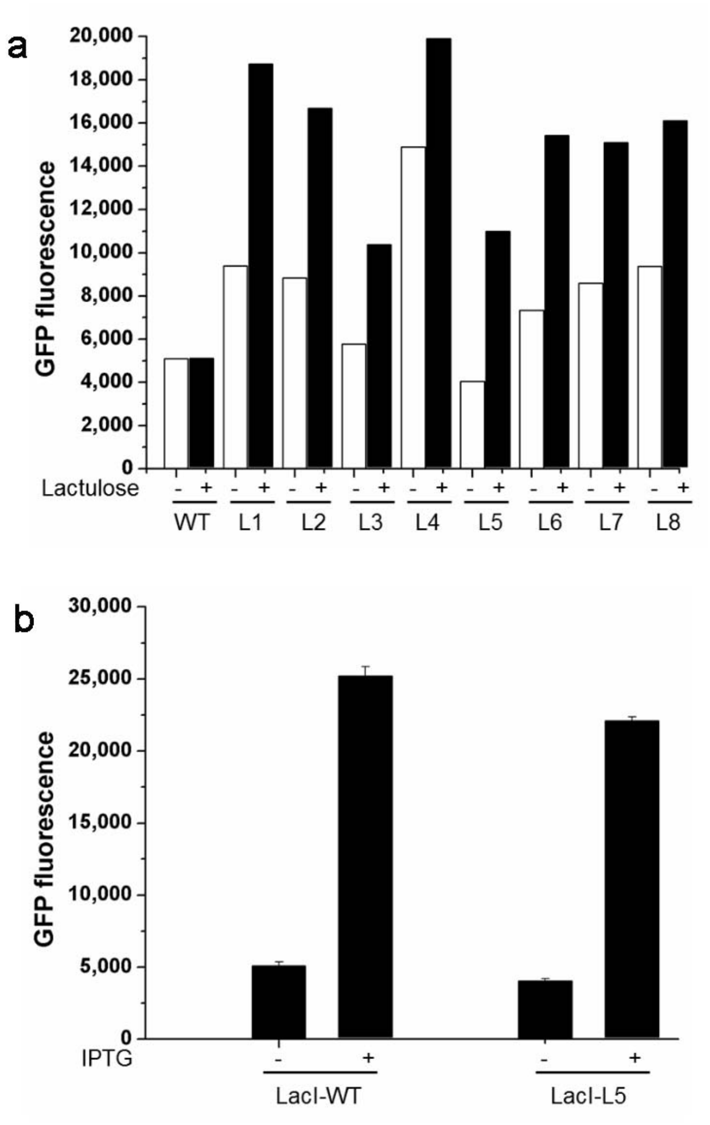


**Figure S1.** (a) GFP fluorescence of strain BLGR harboring plasmid pLac7 expressing wild-type (LacI-WT) or the selected mutant LacIs in the presence (+) and absence (-) of 1 mM lactulose. (b) GFP fluorescence of strain BLGR harboring wild-type LacI (LacI-WT) or LacI-L5 in the presence (+) and absence (-) of 1 mM IPTG.

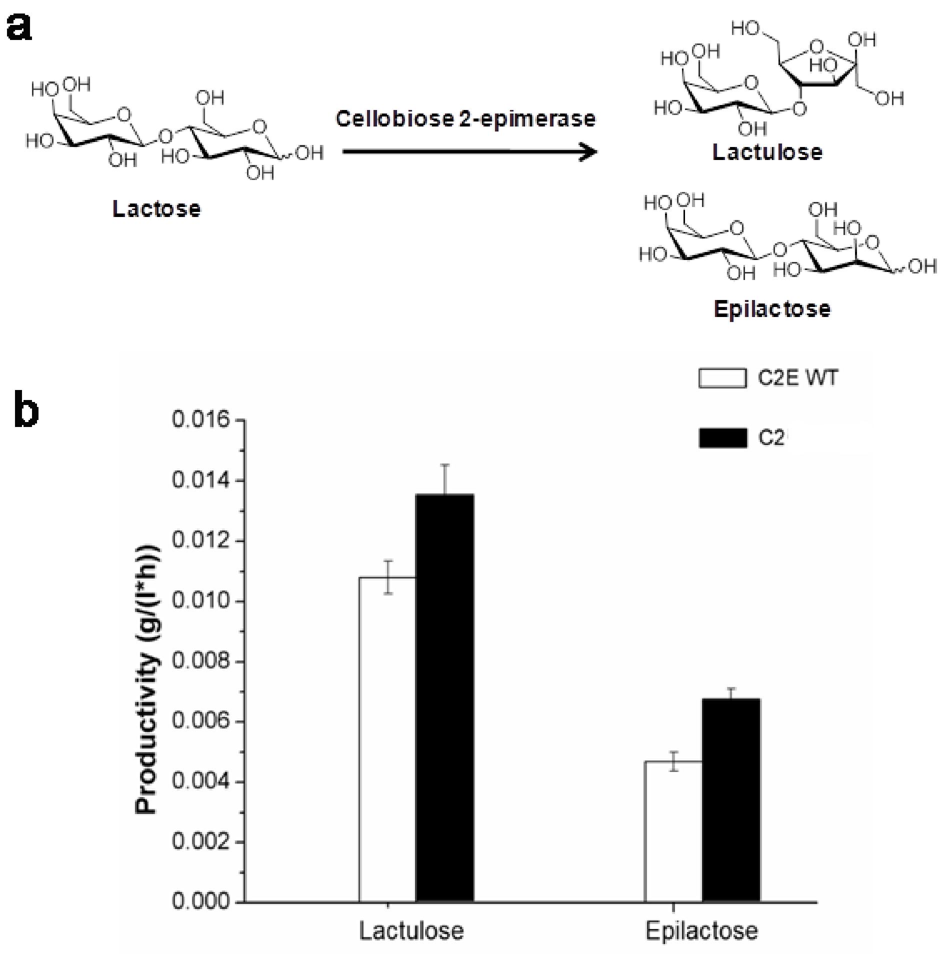


**Figure S2.** (a) Reaction catalyzed by cellobiose 2-epimerase from *Caldicellulosiruptor saccharolyticus* produces lactulose. (b) Productivity of wild-type C2E and mutant C2 in producing lactulose and epilactose at 37$^{o}$C.

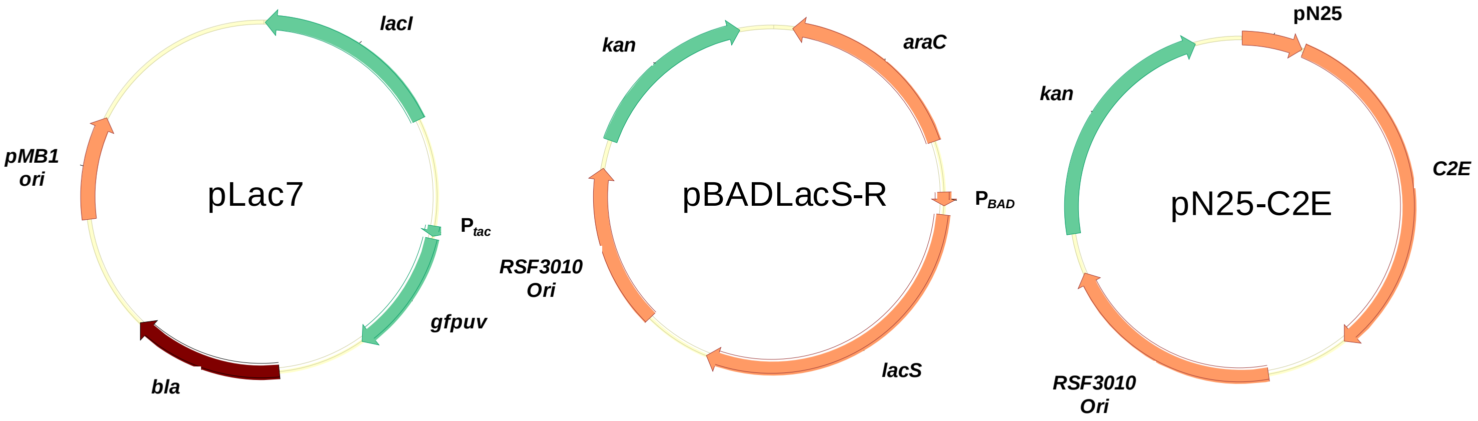


**Figure S3.** Plasmid maps.

**Table S1**. Strains and plasmids used in this study.

| Resource | Relevant characteristics | Source |
|---|---|---|
| **Strains** | | |
| BLGR | BW25113 (Δ*araFGH*, Δ*araBAD*, Δ*lacZ*), with *lacI*, *galK* and *recA* deleted | This study |
| **Plasmids** | | |
| pLac7 | *lacI*; $P_{tac}$-*gfpuv* | This study |
| pLac7-L5 | *lacI-L5*; $P_{tac}$-*gfpuv* | This study |
| pBADLacS | $P_{BAD}$-*lacS*; pBR322 origin; Amp resistance | Jiang, et al., 2015 |
| pBADLacS-R | $P_{BAD}$-*lacS*; RSF3010 origin; Kan resistance | This study |
| pN25-C2E | $P_{PN25}$ controlled *C2E* or *C2E* mutant | This study |
| pBADLacI | $P_{BAD}$ controlled *lacI* or *lacI* mutant | This study |

**Table S2**. Primers used in this study.

| Primer name | Sequence (5'to 3') |
|---|---|
| LacI-I79-for | 5'-GCCACCTCCAGTCTGGCCCTGCACGCGCCGTCGCAANNSGTCGCGG CG -3' |
| LacI-F161-rev | 5'-CGCGTACCGTCTTCATGGGASNNAATAATACTG-3' |
| LacI-F161- for | 5'- TCCCATGAAGACGGTACGCG-3' |
| LacI-N246-rev | 5'- GCGCCCAGCGCCATCTGATCSNNGGCAACCAG-3' |
| LacI-N246-for | 5'-GATCAGATGGCGCTGGGCGC-3' |
| LacI-D296-rev | 5'- CGGTCCACGCTGGTTTGCCCSNNCAGGCGAAA-3' |
| gfp-for-AseI | 5'- ATAGATTAATGGCTAGCAAAGGAGAAGAACTT-3' |
| gfp-rev-XbaI | 5'- AGAGTCTAGA TTATTTGTAGAGCTCATCCATGCCATG-3' |
| C2E-for | 5'-ATGGATATTACAAGGTTTAAG-3' |
| C2E-rev | 5'-TTAGTCAACCCTTTTTATTAT-3' |
| C2E-KpnI-for | 5'-AAGGTACCATGGATATTACAAGGTTTAAG-3' |
| C2E-SacI-rev | 5'-ACGAGCTCGTGGTGGTGGTGGTGGTGGTCAACCCTTTTTATTAT-3' |
| pBADLacS-R-SacI-for | 5'-AAGAGCTCGGTCTGCAGCTGGTGCC-3' |
| pBADLacS-R-KpnI-rev | 5'-AAGGTACCCTCCTGTTAGCCCAAAAAACGG-3' |
| pN25-SphI-for | 5'-ACAGCATGCGAATTCGAGCTCGGTACCC-3' |
| pN25-SpeI-rev | 5'-AACACTAGTATCTCCTTCTTAAAAGATCTTTTGAAT-3' |
| LacI-Ex-NdeI-for | 5'-ACCATATGAAACCAGTAACGTTATACGATGTCGC-3' |
| LacI-Ex-XhoI-rev | 5'-ACCTCGAGTCACTGCCCGCTTTCCAGT-3' |

[a] Restriction sites were underlined.

[b]N: A, C, G or T; S: G or C.
